# A new method for evaluating the rock mass damage index based on the field point load strength

**DOI:** 10.1098/rsos.181591

**Published:** 2019-03-06

**Authors:** Lei Wen, Zhou quan Luo, Shi jiao Yang, Ya guang Qin, Shao wei Ma, Hong Jiang

**Affiliations:** 1 School of Resources and Safety Engineering, Central South University, Changsha 410083, Hunan, People's Republic of China; 2 School of Nuclear Resources Engineering, University of South China, Hengyang 421001, People's Republic of China

**Keywords:** damage index, point load strength, integrality coefficient, P-wave velocity, rock mass

## Abstract

The damage index is a crucial controlling parameter for rock mass strength and deformation in civil, geological and mining engineering projects. In this study, a new method was proposed for computing the damage index of a rock mass based on the field point load strength. This method provides a strength ratio whose numerator is the point load strength (PLS) of splitting along the pre-existing joint and whose denominator is the PLS of intact rock failure. To validate this method, the authors derived a relationship between the damage index and the integrality coefficient using an empirical relation. Moreover, numerous experimental tests were conducted, including P-wave testing and on-site point load testing. Finally, linear regression analysis was performed to analyse the correlation between the new damage index *D*_*R*_ and the integrality coefficient *K*_*V*_. This study demonstrates that the presented new method is sensitive to both plasticity and damage in estimating the damage degree of rock masses in underground excavation and tunnel engineering.

## 1. Introduction

Natural rock mass differs from most other engineering materials, because it is anisotropic and contains discontinuities, such as joint bedding planes, folds, sheared zones and faults, resulting in an internal structure that produces different degrees of damage and destructuration. Moreover, excavation disturbance leads to further damage, resulting in great difficulties in estimating the physical and mechanical properties of a rock mass. Many methods for assessing rock mass properties have been proposed and used in engineering practice, such as the rock quality designation (*RQD*) [1], the rock mass rating (*RMR*) [2,3], the Rock Mass Quality Classification Q-system (Q) [4,5] and the geological strength index (*GSI*) [6,7]. Additionally, several researchers have extensively studied the uniaxial compressive strength ratio $\sigma_{\mathrm{cm}}/\sigma_{c}$ of joint rock mass, and regression analysis has been used to analyse the correlation between the ratio $\sigma_{\mathrm{cm}}/\sigma_{c}$ and *RMR* or *GSI*, as shown in table 1, where $\sigma_{c}$ is the unconfined compressive strength of intact rock and $\sigma_{\mathrm{cm}}$ is the unconfined compressive strength of a rock mass. Kulhawy & Goodman [19] and AASHTO [17] reported the variation of the ratio $\sigma_{\mathrm{cm}}/\sigma_{c}$ with *RQD*. Lian-yang Zhang [18] reported the ratio $\sigma_{\mathrm{cm}}/\sigma_{c}$ versus *RQD* relation for estimating the strength of a jointed rock mass. Several researchers [8,10,12–15,20] reported the ratio $\sigma_{\mathrm{cm}}/\sigma_{c}$ versus *RMR* relation for estimating the uniaxial compressive strength of a jointed rock mass. Hoek *et al*. [16] reported the relationships of the ratio $\sigma_{\mathrm{cm}}/\sigma_{c}$ with *RMR*, *GSI* and *D*, where *D* is the degree of disturbance due to blast damage and stress relaxation. These empirical relations provide a reliable basis for the evaluation of rock mass strength. In practice, the engineering properties of rock mass are nearly impossible to measure directly. Thus, many researchers have described a damaged rock mass based on classification indices, such as *GSI* [7,21–23], *RQD* [18,24–26] and *RMR* [8,9,15,24,27–29].

**Table 1.** Empirical studies. $\sigma_{c}$ is the unconfined compressive strength of intact rock, $\sigma_{\mathrm{cm}}$ unconfined compressive strength of rock mass, *RMR* is rock mass rating, *GSI* geological strength index, *D* factor indicating the degree of disturbance due to blast damage and stress relaxation.

author	empirical relation
Aydan & Dalgic [8]	$\frac{\sigma_{\mathrm{cm}}}{\sigma_{c}}=\frac{RMR}{RMR+6(100-RMR)}$
Yudhbir & Prinzl [9]	$\frac{\sigma_{\mathrm{cm}}}{\sigma_{c}}=e^{\frac{7.65(RMR-100)}{100}}$
Laubscher [10] and Singh *et al*. [11]	$\frac{\sigma_{\mathrm{cm}}}{\sigma_{c}}=\frac{RMR-\begin{array}{c} \mathrm{rating}\;\mathrm{for}\;\sigma_{c} \end{array}}{106}$
Ramamurthy *et al*. [12] and Ramamurthy [13]	$\frac{\sigma_{\mathrm{cm}}}{\sigma_{c}}=e^{\frac{RMR-100}{18.75}}$
Kalamaras & Bieniawski [14]	$\frac{\sigma_{\mathrm{cm}}}{\sigma_{c}}=e^{\frac{RMR-100}{24}}$
Sheorey [15]	$\frac{\sigma_{\mathrm{cm}}}{\sigma_{c}}=e^{\frac{RMR-100}{20}}$
Hoek *et al*. [16]	$\frac{\sigma_{\mathrm{cm}}}{\sigma_{c}}=e^{GSI-100/9-3D\left[\frac{1}{2}\;+\;\frac{1}{6}\left(e^{-\frac{GSI}{15}}-e^{\frac{20}{3}}\right)\right]}$
AASHTO [17]	$\frac{\sigma_{\mathrm{cm}}}{\sigma_{c}}=0.0231RQD-1.32\geq 0.15$
Lian-yang Zhang [18]	$\frac{\sigma_{\mathrm{cm}}}{\sigma_{c}}=10^{0.013RQD-1.34}$

According to the above-mentioned studies, researchers have focused on the determination of the ratio $\sigma_{\mathrm{cm}}/\sigma_{c}$, *RMR*, *GSI* and *RQD*, with the methods used varying among researchers and rock mass types. It is a little difficult to measure the value of $\sigma_{\mathrm{cm}}$ of rock mass (or damaged rock) directly; the ratio $\sigma_{\mathrm{cm}}/\sigma_{c}$ can only be obtained using an empirical relation, as reported by Ramamurthy [30], Singh *et al*. [11] and Singh & Rao [31]. The determination of *RMR* parameters is also relatively complex; these parameters include rock strength, *RQD* value, joint space, joint condition and groundwater. *GSI* characterizes the degree to which the strength of a rock mass is weakened under various geological conditions, and is used to describe the characteristics of rock masses in detail. The *GSI* parameter is essentially a qualitative parameter that includes six factors: joints distribution, block shape, the degree of geological disturbance, joint roughness, the weathering degree of the joints and filling situation. The determination of the *RQD* value depends mainly on the drilling technology and mechanical equipment used. In research, the uniaxial compressive strength ratio $\sigma_{\mathrm{cm}}/\sigma_{c}$, *RMR*, *GSI* and *RQD* are the parameters available for describing rock mass properties. However, the method for determining these indices is relatively complicated and tedious, and some indices are only qualitatively estimated based on experience. Thus, in this paper, a new method is proposed for determining the rock mass damage index based on field point load test results.

Field point load tests are preferred because they have strong applicability, as they are flexible in simple testing. Hence, this paper focuses on the determination of the field point load strength ratio and its utilization for evaluating the engineering properties (mainly damage index and integrality coefficient) of rock masses. This new damage index is a point load strength ratio whose numerator is the point load strength (PLS) of splitting along the pre-existing joint and whose denominator is the PLS of the failure of intact rock. One of the critical objectives is to find samples of splitting along a pre-existing joint in terms of significant joint properties of a failure surface. Next, a series of theoretical derivations of the relationship between the field point load strength ratio and the integrality coefficient are presented and briefly discussed. Finally, a new method for calculating the damage index is proposed to determine the damage index of a rock mass. The index can be used for estimating the elastic modulus and unconfined compressive strength of rock masses. To validate this method, various experimental designs and analyses are provided in §§3–5; the discussion and conclusion are presented in §§6 and 7, respectively. This paper outlines the key aspects involved in determining the field point load strength ratio and provides useful information for effectively calculating the integrality coefficient or damage index of rock masses.

## 2. Field point load strength

**Figure 1.**
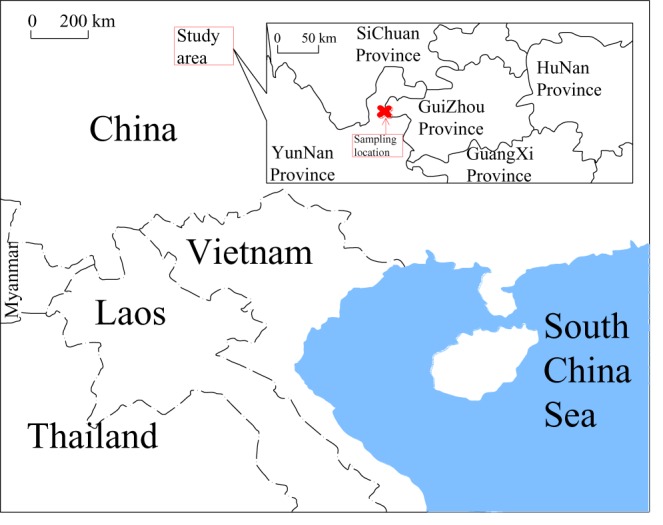
Study area.

The point load strength test has been regarded as an inexpensive and effective testing method for the estimation of the strength of rocks because of its ease of testing, simplicity of specimen preparation and potential field applications [32–35]. The test has been referred to as an indirect method for assessing the tensile or compressive strength of rocks [36]. Some researchers have attempted to establish empirical relations between the uniaxial compressive strength (UTS)/Brazilian tensile strength (BTS) and the point load strength in applying the point load test to various rock types [32,33,37]. In this study, point load strength tests were performed on irregular rock blocks using a digital point load test system according to the guidelines of the ASTM [38]. The study site was the Huize lead and zinc mine, straddling the provinces of YunNan and GuiZhou in southwest China, as shown in figure 1.

**Figure 2.**
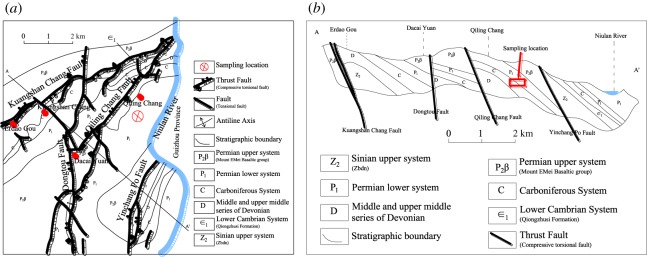
Stratigraphic and geological structure.

Four types of samples of dolomite and limestone were adopted in this test; these four samples originate from different stratigraphic locations, as shown in figure 2. Study area is composed of the northeast to southwest fold and fault, forming the anticline. The sampling location is located between the Qi-lin Chang fault and the Yin-chang Po fault. All samples belong to Palaeozoic strata. The four types of rock are referred to as C_1d_, C_1b_, C_2w_ and P_1q+m_. C_1d_, C_1b_ and C_2w_ belong to the Carboniferous system. P_1q+m_ belongs to the Permian system. C_1d_ is dark-grey and grey cryptocrystalline limestone. C_1b_ is a shallow, pale and flesh-pink coarse-grain dolomite. C_2w_ is light-grey to dark-grey limestone and dolomitic limestone. P_1q+m_ is dark-grey to light-grey, thin-mouth to cryptocrystalline limestone and dolomitic limestone. The physical and mechanical parameters of all rock samples employed are presented in table 2, where *V*_*p*_ is the P-wave velocity of each rock sample.

**Table 2.** Physical and mechanical parameters of rock samples.

								UCS (MPa)			BTS (MPa)		
no.	geological age	rock type	test times	elastic modulus (GPa)	density (t m^−3^)	*V_p_* (m s^−1^)	Poisson's ratio	mean	max.	min.	mean	max.	min.
C_1d_	Carboniferous	limestone	10	30.4	2.70	5344	0.28	69.9	88.34	56.12	4.87	7.879	3.721
C_1b_	Carboniferous	dolomite	10	25.13	2.72	4744	0.25	59.52	75.85	44.86	4.08	5.243	2.991
C_2w_	Carboniferous	limestone	10	29.98	2.71	5130	0.24	75.95	124.74	32.13	5.43	6.53	3.873
P_1q+m_	Permian	limestone	10	21.13	2.73	5514	0.25	60.69	86.62	49.34	4.44	5.653	2.797

**Figure 3.**
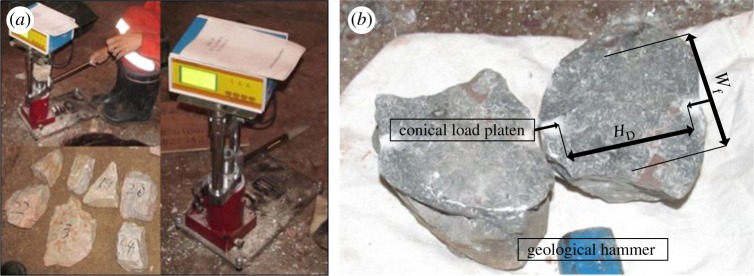
Point load test process: test process, samples and tester (*a*) and sample failure surface (*b*).

The point load tester used in this study consisted of a small hydraulic pump, a hydraulic jack, a pressure gauge and an interchangeable testing frame with a very high transverse stiffness. Panek *et al*. [39] elaborate the detailed process of point load test. In this paper, the details of the technique of field test are carried out as shown in figure 3. The thickness of the irregular rock blocks ranged from 30 to 70 mm, and their lengths were less than 150 mm, as shown in figure 3. All of the point load tests were performed on irregular rock blocks from four different types of sedimentary rock. Each irregular rock block was slowly loaded until failure. According to the failure mode, the measured results could be divided into two failure types based on visual inspection alone: intact rock failure in which no apparent joint phenomenon could be observed on the failure surface of the samples, and splitting along a pre-existing joint. When it was not clear whether a joint occurred on a sample failure surface, the sample was excluded. Thus, a total of 193 irregular block samples were obtained. The raw data of all specimens are presented in table 3, where *L*, *W*, *H* are the maximum length, width and height of the irregular samples before testing, respectively; *P* is the maximum load applied during loading; $W_{f}$ is the effective width of the fracture surface; and *H*_*D*_ is the distance between the loading points; the letter ‘I’ represents samples exhibiting intact rock failure; and the letter ‘J’ represents samples exhibiting splitting along a pre-existing joint.

**Table 3.** The test parameters of point load strength. *L*, *W*, *H* are the maximum length, width and height of failure surface, the letter ‘I’ represents samples exhibiting intact rock failure; and the letter ‘J’ represents samples exhibiting splitting along a pre-existing joint, *P* is the maximum load applied during loading; $W_{f}$ is the effective width of the fracture surface; and *H*_*D*_ is the distance between the loading points.

	size (mm)							
no.	*L*	*W*	*H*	*P* (kN)	*H_D_* (mm)	*W*_f_ (mm)	*PLS*	failure modes
C_1d_ is dark grey and grey cryptocrystalline limestone								
1	95	61	41	16.17	36	75	4.70	I
2	80	64	38	13.52	34	55	5.67	I
3	116	61	48	22.98	35	94	5.48	I
4	125	75	36	13.47	38	72	3.86	I
5	81	63	35	38.48	33	75	1.22	J
6	115	65	40	8.88	39	76	2.35	J
7	71	59	59	20.59	58	70	3.98	J
8	107	86	47	24.14	41	83	5.57	I
9	120	73	43	28.80	45	74	2.79	J
10	89	65	30	8.71	31	79	6.78	I
11	79	46	33	10.03	31	40	6.35	I
12	95	69	31	9.44	31	69	3.47	I
13	96	65	31	10.97	36	88	2.72	I
14	140	85	45	16.98	48	141	1.97	I
15	101	84	65	10.16	60	92	1.45	J
16	95	70	37	16.49	39	98	3.39	I
17	100	80	38	8.86	29	66	3.63	I
18	103	74	36	18.79	35	94	4.48	I
19	101	65	37	16.71	42	92	3.40	I
20	113	61	57	15.52	58	72	4.37	J
21	105	91	46	22.21	42	95	4.37	I
22	90	53	40	21.45	40	61	6.90	I
23	74	53	39	14.93	38	51	6.05	I
24	110	79	38	3.98	43	58	1.25	J
25	105	46	34	9.06	34	45	4.65	J
26	135	93	69	24.49	65	115	2.57	I
27	130	82	30	5.22	30	92	1.48	J
28	140	49	34	11.18	30	75	3.90	I
29	135	81	52	17.27	58	86	2.72	J
30	106	87	58	22.94	50	112	3.22	I
31	110	80	41	13.91	32	110	3.10	J
32	67	65	33	13.60	29	49	7.51	I
33	76	61	45	25.52	39	65	7.90	I
34	115	65	32	5.10	29	62	2.23	J
35	78	55	47	20.72	34	71	6.74	I
36	105	90	37	9.37	29	77	3.29	J
37	77	70	45	14.23	40	64	4.36	I
38	76	60	50	17.30	29	81	5.78	I
39	130	75	56	20.64	54	105	2.86	I
40	90	63	45	19.60	36	72	5.77	I
41	133	96	40	10.92	39	133	1.65	J
42	129	91	44	17.86	49	87	3.29	I
43	165	92	39	7.95	37	85	1.98	J
44	136	66	52	21.66	48	62	5.71	I
45	165	79	43	15.76	30	96	4.29	I
46	128	84	34	8.98	38	87	2.13	J
47	123	92	42	17.73	45	110	2.81	I
48	129	99	46	21.18	38	93	4.70	I
49	118	89	51	22.21	59	89	3.32	I
50	145	82	61	20.40	60	115	2.32	I
52	104	88	42	12.56	40	88	2.80	I
53	136	94	45	15.74	47	96	2.74	I
54	91	68	53	16.28	54	88	2.69	I
55	128	89	51	12.67	51	106	1.84	J
56	116	83	53	22.82	51	118	2.98	I
57	110	86	42	26.12	37	106	5.23	I
58	145	96	34	20.51	29	132	4.21	I
C_2w_ is light grey to dark grey limestone and dolomitic limestone								
1	90	75	39	17.32	39	91	3.83	I
2	96	84	49	12.35	55	61	2.89	J
3	146	100	49	24.87	49	99	4.02	I
4	98	79	34	35.54	38	101	7.27	I
5	104	97	35	16.12	39	98	3.31	I
6	92	87	48	12.23	49	75	2.61	J
7	145	91	34	11.97	33	105	2.71	J
8	112	67	36	20.42	38	71	5.94	I
9	105	92	35	19.96	41	104	3.67	I
10	137	101	45	41.85	41	90	8.90	I
11	125	79	34	17.19	34	61	6.51	I
12	114	109	49	25.77	56	75	4.82	I
13	114	89	49	17.92	49	104	2.76	J
14	133	90	57	13.50	50	101	2.10	J
15	114	84	52	15.92	51	107	2.29	J
16	131	93	59	24.93	55	92	3.87	I
17	135	97	57	35.12	58	114	4.17	J
18	95	56	35	2.53	39	30	1.7	J
19	125	93	40	11.02	39	95	2.33	J
P_1q+m_ is dark grey to light grey cryptocrystalline limestone								
1	123	83	35	8.41	36	68	2.70	J
2	134	94	49	21.83	38	80	5.64	I
3	98	75	43	11.56	42	71	3.04	I
4	120	67	31	11.56	25	82	4.43	I
5	134	84	45	20.13	44	79	4.55	I
6	135	93	63	29.34	59	88	4.44	I
7	120	98	42	16.00	50	97	2.59	I
8	105	82	51	20.86	44	57	6.53	I
9	121	56	30	11.35	27	79	4.18	I
10	77	55	35	15.30	23	94	5.64	I
11	99	94	34	10.35	38	94	2.27	J
12	120	94	33	5.74	34	115	1.15	J
13	94	67	46	12.61	38	64	4.07	I
14	109	89	53	20.37	53	91	3.32	I
15	108	93	34	13.13	34	86	3.53	J
16	125	85	43	20.08	39	99	4.08	I
17	135	80	44	16.33	38	79	4.27	I
18	85	79	44	11.23	33	75	3.56	I
19	86	61	37	12.27	33	66	4.42	I
20	95	50	32	15.25	32	50	7.48	I
21	125	95	51	23.26	47	115	3.38	I
22	130	98	51	13.28	49	92	2.31	J
23	105	94	38	13.44	38	90	3.08	I
24	121	62	46	3.50	41	102	0.66	J
25	131	89	40	10.21	31	64	4.04	J
26	115	79	42	15.82	30	78	5.31	I
27	123	90	41	14.10	36	84	3.66	I
28	130	59	41	17.78	45	63	4.92	I
29	120	95	46	11.80	40	93	2.49	J
30	100	86	43	5.71	29	72	2.15	J
31	135	103	52	24.45	42	103	4.44	I
32	98	69	33	5.79	33	67	2.05	I
33	125	76	41	28.90	42	94	5.74	I
34	105	42	47	22.56	38	65	7.17	I
35	105	72	38	11.16	30	97	3.01	I
36	95	60	42	15.28	32	81	4.63	I
37	150	54	35	11.34	36	53	4.66	I
38	125	65	37	4.31	28	54	2.24	J
39	98	65	50	8.17	31	93	2.23	I
40	88	67	50	22.78	48	85	4.38	I
41	130	85	45	5.34	42	85	1.17	J
42	77	65	47	13.21	39	76	3.50	I
43	125	85	30	6.81	24	42	5.30	J
44	105	64	43	13.74	46	57	4.11	I
45	100	65	57	31.61	53	70	6.69	I
46	75	64	50	21.78	42	82	4.97	I
47	94	65	28	14.98	25	81	5.81	I
48	155	95	37	21.50	24	115	6.11	I
49	110	75	50	5.23	43	53	1.81	J
50	120	97	50	8.43	40	98	1.69	J
51	143	90	55	37.14	48	100	6.07	I
52	95	48	45	9.13	44	67	2.43	I
C_1b_ is shallow pale and flesh pink coarse-grain dolomite								
1	77	57	45	10.14	40	91	2.19	I
2	76	73	38	13.83	33	53	6.21	I
3	79	69	42	13.80	43	82	3.07	I
4	122	48	38	3.63	32	79	1.13	J
5	61	46	39	2.77	37	62	0.95	J
6	110	86	39	6.93	39	86	1.62	J
7	96	62	42	12.15	42	76	2.99	J
8	89	64	37	6.34	40	61	2.04	I
9	112	75	54	16.97	46	71	4.08	I
10	77	62	35	21.02	32	65	7.93	I
11	85	65	33	14.50	32	42	8.47	I
12	93	75	38	1.98	35	51	3.51	J
13	91	57	42	11.72	42	65	3.37	I
14	105	49	32	5.02	32	65	1.90	J
15	87	69	36	9.59	39	85	2.27	J
16	113	81	43	4.04	44	115	0.63	J
17	105	79	34	5.79	34	95	1.41	J
18	109	42	34	4.04	32	101	0.98	J
19	75	65	45	9.81	52	74	2.00	J
20	115	46	34	5.72	32	52	2.70	I
21	116	73	32	7.08	31	94	1.91	J
22	89	73	45	22.34	34	67	7.70	I
23	81	46	44	6.79	44	49	2.47	I
24	95	49	39	1.99	38	102	0.40	J
25	109	77	33	15.63	40	95	3.23	I
26	97	74	31	14.09	38	105	2.77	I
27	104	98	47	30.73	47	96	5.35	I
28	117	76	39	11.24	41	75	2.87	J
29	82	76	33	10.68	36	73	3.19	J
30	115	75	33	20.48	33	65	7.49	I
31	92	86	44	15.76	41	59	5.11	I
32	105	76	46	15.96	43	97	3.00	I
33	128	48	33	13.74	33	65	5.03	I
34	94	75	43	7.23	46	89	1.39	J
35	68	47	30	8.65	25	47	5.78	I
36	98	72	59	28.87	58	59	6.62	I
37	91	48	45	18.69	42	51	6.85	I
38	135	80	40	18.84	40	110	3.36	I
39	120	85	50	6.52	50	75	1.36	I
40	125	80	37	8.95	40	120	1.46	I
41	135	85	60	15.49	55	125	1.77	J
42	135	85	55	14.15	40	50	5.55	I
43	110	75	37	9.73	35	120	1.82	J
44	100	90	30	11.97	30	90	3.48	I
45	100	85	60	20.84	55	85	3.50	I
46	105	67	38	6.65	40	35	3.73	J
47	115	85	45	11.88	40	85	2.74	J
48	100	65	38	13.98	40	67	4.09	I
49	130	95	58	9.71	50	88	1.73	J
50	108	10	47	7.60	50	100	1.19	J
51	90	75	33	5.71	35	45	2.84	J
52	100	55	43	12.30	40	64	3.77	I
53	110	78	48	8.00	40	105	1.49	J
54	105	76	63	27.51	65	58	5.73	J
55	107	60	45	16.62	40	94	3.47	J
56	108	55	46	10.35	51	60	2.66	I
57	100	85	46	13.99	50	100	2.20	J
58	96	85	44	13.99	34	96	3.37	I
59	139	50	50	16.41	42	48	6.39	I
60	103	39	25	11.73	27	82	4.16	I

Irregular rock blocks must be corrected to the standard equivalent diameter (*D*_*e*_) of 50 mm. Size correction can be performed graphically and mathematically, as suggested by ASTM [38] procedures. The point load index PLS is determined by the following equations:

2.1 $$D_{e}=\sqrt{\frac{4H_{D}W_{f}}{\pi }}$$

and

2.2 $$\mathrm{PLS}=\frac{4P}{\pi D_{e}^{2}},$$

where *P* is the failure load and *D*_*e*_ is the equivalent diameter of irregular blocks; *H*_*D*_ and $W_{f}$ are of the maximum length and average width of the failure surface in millimetres, respectively (figure 3). As shown in table 3, the fluctuation range of PLS value is larger. The main reason for the large fluctuation range of PLS value is the irregularity of rock sample and the different degree of micro-cracks in the sample. According to the results shown in table 3 and table 4, the PLS value of C_1d_ fluctuated between 1.22 and 7.9 MPa, with mean values of 2.36 MPa in cases of splitting along a pre-existing joint and 4.41 MPa in cases of intact rock failure. The PLS value of C_1b_ was found to vary in a broad range (0.63–7.93 MPa), with a mean value of 2.56 MPa, a mean value of 2.14 MPa for splitting along a pre-existing joint and a mean value of 4.38 MPa for intact rock failure. The PLS values of C_2w_ were found to be in the range 0.66–7.48 MPa, with a mean value of 3.44 MPa, a mean value of 2.39 MPa for splitting along a pre-existing joint and a mean value of 4.49 MPa for intact rock failure. The PLS value of P_1q+m_ fluctuated between 2.1 and 8.9 MPa, with a mean value of 4.22 MPa, a mean value of 2.46 MPa for splitting along a pre-existing joint and a mean value of 5.21 MPa for intact rock failure.

**Table 4.** Average values of PLS on different rock types.

	C_1d_			C_1b_			C_2w_			P_1q+m_		
	limestone			dolomite			limestone			limestone		
rock type	J	I	all	J	I	all	J	I	all	J	I	all
number	18	40	58	27	33	60	14	38	52	7	10	17
mean	2.36	4.41	3.75	2.14	4.38	2.56	2.39	4.49	3.44	2.46	5.21	4.22
s.d.	2.19	1.356	1.665	1.32	1.938	1.97	1.818	1.341	1.598	0.59	1.91	1.85

In previous studies, samples with splitting along a pre-existing joint were excluded from the set of tested samples [37,40]. In this study, the results of all point load tests were recorded clearly. Based on all of the test results collected, a new conclusion is drawn. The sum of the ratio $\mathrm{PL}S_{\mathrm{sm}}/\mathrm{PL}S_{s}$ and the integrality coefficient *K*_*V*_ is approximately equal to 1, where $\mathrm{PL}S_{\mathrm{sm}}$ is the PLS of rock samples with splitting along a pre-existing joint, $\mathrm{PL}S_{s}$ is the PLS of samples with intact rock failure and *K*_*V*_ is the integrality coefficient. The integrality coefficient of a rock mass is determined as suggested by GB50218–94 [41] in China and is expressed as follows:

2.3 $$K_{V}=\frac{V_{P}^{{\;}^{\prime }2}}{V_{p}^{2}},$$

where *V*_*P*_ is the P-wave velocity of intact rock and $V_{p}^{\prime }$ is the P-wave velocity of a rock mass.

The new damage index *D*_*R*_ can be defined as the ratio of the value for splitting along a pre-existing joint $I_{sm}$ to the value for intact rock failure $\mathrm{PL}S_{s}$ (this sentence explains equation (2.4)). The new damage index *D*_*R*_ is expressed as follows:

2.4 $$D_{R}=\frac{\mathrm{PL}S_{\mathrm{sm}}}{\mathrm{PL}S_{s}},$$

where $\mathrm{PL}S_{\mathrm{sm}}$ is the PLS of rock samples with splitting along a pre-existing joint, $\mathrm{PL}S_{s}$ is the PLS of samples with intact rock failure.

The field point load strength ratio is presented in table 5. These results show that the integrality coefficient *K*_*V*_ is approximately equal to the difference 1 − *D*_*R*_, and the deviation between *K_V_* and the difference 1 − *D*_*R*_ is also listed in table 5. To verify this conclusion, a theoretical derivation is conducted in §3 and the rock mass joint statistics of the study area and the field experimental tests are derived in §4.

**Table 5.** Calculated *D*_*R*_ value based on field point load strength.

name	C_1d_	C_1b_	C_2w_	P_1q+m_
*D*_*R*_	0.535	0.489	0.532	0.472
1 − *D*_*R*_	0.465	0.511	0.468	0.528
*K*_*V*_	0.453	0.496	0.481	0.517
deviation between *K*_*V*_ and 1 − *D*_*R*_	2.24%	3.1%	2.4%	2.3%

## 3. Theoretical derivation of integrity and damage index

### 3.1. Damage index and elastic modulus

Because a rock mass is a complex and heterogeneous material, its damage level cannot be directly measured by current methods. In other words, it is difficult to measure the damage area of damaged rock. However, the strength of damaged rock can be accurately measured in a mechanics laboratory or by *in situ* tests. In this respect, a new method for calculating the damage index of a rock mass is proposed via *in situ* point load strength testing, as formulated in equation (2.4).

According to Lemaitre [42], the definition of the damage variable *D* is different from the new damage index *D*_*R*_ in equation (2.4). It is assumed that the newly defined *D*_*R*_ is reasonable and it can replace the damage variable *D* of Lemaitre's [42] definition. Like that, the newly defined *D*_*R*_ is tried to derive a series of theoretical relationships. Finally, the assumption is verified by field point load strength test.

Based on the stress–strain relationship of intact rock under uniaxial compressive testing, the elastic strain *ɛ_r_* of intact rock is represented as follows:

3.1 $$\varepsilon_{r}=\frac{\sigma_{c}}{E},$$

where $\sigma_{c}$ and *E* are the uniaxial compressive strength (UCS) and the elastic modulus of the intact rock, respectively.

According to Lemaitre [42], the relationship between the elastic modulus *E* of intact rock and the elastic modulus *E*_*m*_ of a rock mass can be expressed as follows:

3.2 $$E_{m}=\frac{E}{1-D_{R}}.$$

Based on the equivalent stress proposed by Lemaitre [42], it is assumed that the deformation of a rock mass can be represented by the equivalent stress. The elastic strain $\varepsilon_{m}$ of a rock mass can be expressed as follows:

3.3 $$\varepsilon_{m}=\frac{\sigma_{\mathrm{cm}}}{E}=\frac{\sigma_{c}}{E(1-D_{R})}=\frac{\sigma_{c}}{E_{m}},$$

where *E*_*m*_ is the elastic modulus of the rock mass, then

3.4 $$E_{m}\varepsilon_{m}=E_{r}\varepsilon_{r}.$$

### 3.2. Damage index and integrality coefficient

A rock mass is composed of statistically distributed joints/fracture and intact rock blocks. Hence, intact rock can be regarded as a homogeneous material, and a rock mass or damaged rock can be regarded as a heterogeneous material. Based on the theory of elastic waves, it is well known that the P-wave velocity *V*_*p*_ in homogeneous material (intact rock) is expressed as follows:

3.5 $$V_{p}=\sqrt{\frac{E_{r}^{\prime }(1-\mu )}{\rho (1+\mu )(1-2\mu )}},$$

where *V*_*p*_, $E_{r}^{\prime }$, μ and *ρ* are the P-wave velocity, dynamic modulus of elasticity, Poisson's ratio and density of the intact rock, respectively. The P-wave velocity $V_{p}^{\prime }$ in heterogeneous material (rock mass or damaged rock) is expressed as follows:

3.6 $$V_{p}^{\prime }=\sqrt{\frac{E_{m}^{\prime }(1-\mu^{\prime })}{\rho^{\prime }(1+\mu^{\prime })(1-2\mu^{\prime })}},$$

where $V_{p}^{\prime }$, $E_{m}^{\prime }$, $\mu^{\prime }$ and $\rho^{\prime }$ are the P-wave velocity, dynamic modulus of elasticity, Poisson's ratio and density of the joint rock mass or damaged rock mass, respectively.

Palmstrom & Singh [43] proposed that the ratio of the static modulus of elasticity to the dynamic modulus of elasticity of intact rock is equal to that of a damaged rock or rock mass. The function is expressed as follows:

3.7 $$\frac{E_{m}}{E_{m}^{\prime }}=\frac{E_{r}}{E_{r}^{\prime }}.$$

In combination, equations (3.2) and (3.7) can be expressed as follows:

3.8 $$E_{m}^{\prime }=E_{r}^{\prime }(1-D_{R}).$$

From the point of view of rock engineering, Poisson's ratio and density of a rock mass and those of a rock block are approximately the same. The properties are expressed as follows:

3.9 $$\rho^{\prime }=\rho$$

and

3.10 $$\mu^{\prime }=\mu .$$

Therefore, the new damage index *D*_*R*_ can be expressed as follows:

3.11 $$D_{R}=1-\frac{V_{p}^{{\;}^{\prime }2}}{V_{p}^{2}}.$$

Equation (3.11) can also be expressed as follows:

3.12 $$D_{R}+K_{V}=1,$$

where *K*_*V*_ is the integrality coefficient of the rock mass, as indicated in equation (2.3).

The results show that the integrality coefficient is equal to 1 and the damage index is 0 in intact rock. When the rock is absolutely destroyed, the integrality coefficient is 0, and the damage index is 1. In practical engineering, a rock mass is variably damaged under the effects of complex geologic and engineering activities; thus, practically no absolute intact rock occurs in nature.

## 4. Experimental scheme and results

To verify the new discovery discussed in §2 and the theoretical derivation presented in §3, experiments were performed and are detailed in this section; these include a field point load test and a P-wave velocity test.

### 4.1. Point load test results

To verify the results presented in table 5, 15 testing sites were prepared for measuring the field point load strength and P-wave velocity in each lithology of the strata. When the *in situ* point load test samples were collected, some blocks of irregular joints were also gathered deliberately. A total of 30 samples per test site were prepared to conduct point load strength tests; the test method is discussed in §2. The measured results were recorded clearly for the average values of PLS for intact rock failure and splitting along a pre-existing joint, as listed in table 6, where the letter ‘I’ represents samples with intact rock failure, the letter ‘J’ represents samples with splitting along a pre-existing joint, *n* is the number of effective trials performed at each testing site and *N* is the total number of samples at each testing site.

**Table 6.** The average values of PLS on different rock types and different failure types.

	C_1d_			C_1b_			C_2w_			P_1q+m_		
rock type	*n/N*	I	J	*n/N*	I	J	*n/N*	I	J	*n/N*	I	J
Test 1	24/30	3.29	1.22	21/30	3.37	1.95	26/30	1.61	0.92	25/30	3.83	1.29
Test 2	26/30	2.32	2.35	27/30	7.93	2.19	25/30	2.45	1.78	24/30	4.02	2.61
Test 3	23/30	2.80	2.72	23/30	2.70	1.84	24/30	4.23	1.50	23/30	7.27	3.51
Test 4	25/30	2.74	1.79	25/30	3.51	1.56	21/30	3.57	1.68	25/30	5.94	2.10
Test 5	27/30	2.69	1.44	24/30	3.23	1.78	22/30	4.78	2.43	26/30	3.67	2.71
Test 6	22/30	3.84	3.39	22/30	2.77	1.41	25/30	3.02	0.89	23/30	4.82	1.92
Test 7	25/30	2.98	2.63	25/30	3.19	0.98	24/30	2.41	1.69	27/30	2.67	0.81
Test 8	26/30	4.70	3.2	26/30	5.11	2.00	25/30	1.21	0.54	22/30	8.90	3.87
Test 9	24/30	3.86	2.62	23/30	5.03	1.91	27/30	2.45	0.97	23/30	6.51	2.69
Test 10	21/30	6.90	1.25	25/30	5.78	2.40	24/30	2.02	0.91	26/30	2.29	1.34
Test 11	27/30	4.48	1.48	27/30	2.88	1.23	26/30	3.72	2.05	25/30	4.71	2.57
Test 12	23/30	4.37	2.90	25/30	3.36	1.77	23/30	3.65	2.31	22/30	3.31	1.63
Test 13	26/30	5.50	2.72	22/30	3.48	1.82	23/30	1.71	0.65	25/30	5.24	3.01
Test 14	25/30	2.57	1.65	23/30	4.09	1.73	25/30	2.97	0.94	24/30	4.28	1.15
Test 15	22/30	3.32	1.98	26/30	3.77	2.74	27/30	2.78	1.42	21/30	3.59	2.43

### 4.2. P-wave velocity test results

Ultrasonic methods are non-destructive and relatively easy to use, both in the field and in the laboratory. The P-wave velocity has been used to characterize rock masses by many researchers [44–49]. In this study, the P-wave velocity of rock masses and that of intact rock were measured to calculate the integrality coefficient of the rock masses. The P-wave velocity of the rock masses was determined in the field, while that of intact rock was performed in the laboratory.

**Figure 4.**
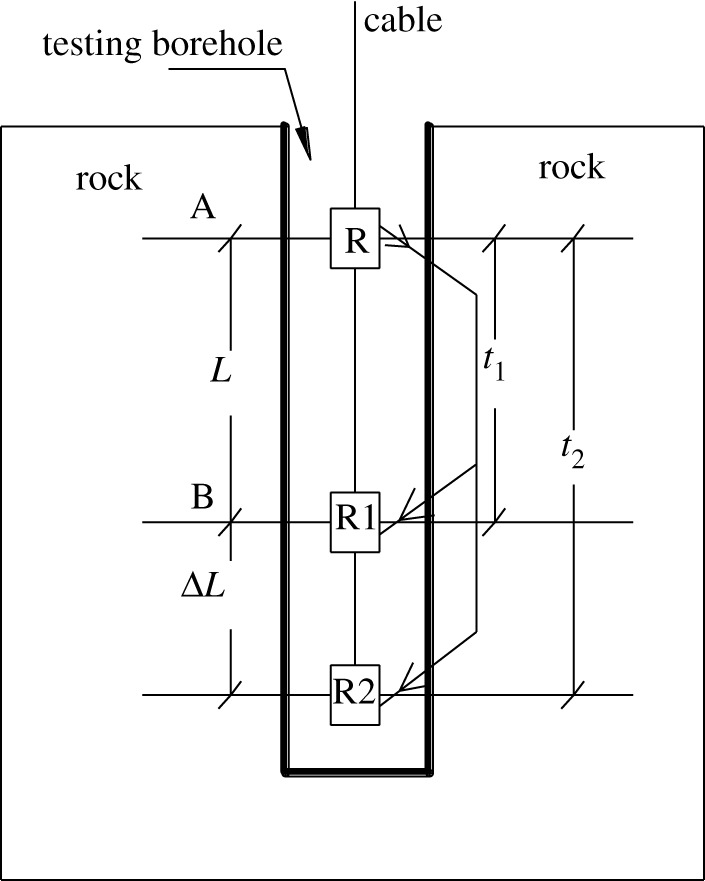
Single-borehole method for measuring P-wave velocity.

The P-wave velocity of the rock masses was measured by a single-borehole testing method using an RSM-SY5(T) intelligent engineering instrument. The single-borehole P-wave velocity testing method is illustrated in figure 4.

As shown in figure 4, a launching transducer and two receiving transducers are placed in the borehole, with pure water used as the coupling medium. A P-wave is launched from transducer R and received by transducers R1 and R2. The P-wave propagates into the rock mass, through the water and along the wall of the borehole. The P-wave arrives at receiving transducer R1 at time *t*_1_ and at receiving transducer R2 at time *t*_2_. Therefore, the P-wave velocity between R1 and R2 (shown in figure 4) can be calculated as

4.1 $$\Delta t=t_{2}-t_{1}$$

and

4.2 $$V_{p}^{\prime }=\frac{\Delta L}{\Delta t},$$

where $V_{p}^{\prime }$ is the P-wave velocity of the rock mass and ΔL is the P-wave propagation distance between receiving transducers R1 and R2.

The P-wave velocity of each rock mass was measured five times for each measuring point. The mean P-wave velocities are listed in table 7, where N_1_ represents the number of times each sample was tested. The P-wave velocity of each rock mass was found to occupy a very broad range (2598.40−4580.27 m s^−1^), varying widely between different types of rock mass and different test points. For the intact rock tested in the laboratory, the P-wave velocity varied from 3514 m s^−1^ to 6075 m s^−1^, and its mean value was found to occupy a very broad range (4744−5514 m s^−1^).

**Table 7.** The average values of P-wave velocity in rock mass and intact rock samples.

	C_1d_		C_1b_		C_2w_		P_1q+m_	
rock type	N_1_	P-velocity (m s^–1^)	N_1_	P-velocity (m s^–1^)	N_1_	P-velocity (m s^–1^)	N_1_	P-velocity (m s^–1^)
Test 1	5	4243.31	5	3284.80	5	3146.81	5	4376.60
Test 2	5	3562.66	5	4261.30	5	2598.40	5	3354.04
Test 3	5	3520.99	5	2991.28	5	3765.43	5	4014.25
Test 4	5	3121.02	5	3906.89	5	3518.24	5	4341.73
Test 5	5	3724.68	5	3441.31	5	3354.51	5	2917.73
Test 6	5	3955.28	5	3554.17	5	4053.28	5	4306.57
Test 7	5	3121.02	5	4199.09	5	2683.61	5	4546.96
Test 8	5	3024.97	5	3973.68	5	3146.81	5	4089.29
Test 9	5	3214.21	5	4071.81	5	3612.92	5	4126.30
Test 10	5	4577.93	5	3873.07	5	3643.94	5	3573.48
Test 11	5	4346.40	5	3906.89	5	3074.46	5	3615.77
Test 12	5	3073.37	5	3479.34	5	2846.40	5	3898.99
Test 13	5	3841.71	5	3591.00	5	3795.20	5	3657.57
Test 14	5	3349.13	5	3940.43	5	3969.12	5	4580.27
Test 15	5	3955.28	5	2856.26	5	3286.74	5	3262.13
intact rock P-wave velocity	5344 m s^−1^		5130 m s^−1^		4744 m s^−1^		5514 m s^−1^	

**Figure 5.**
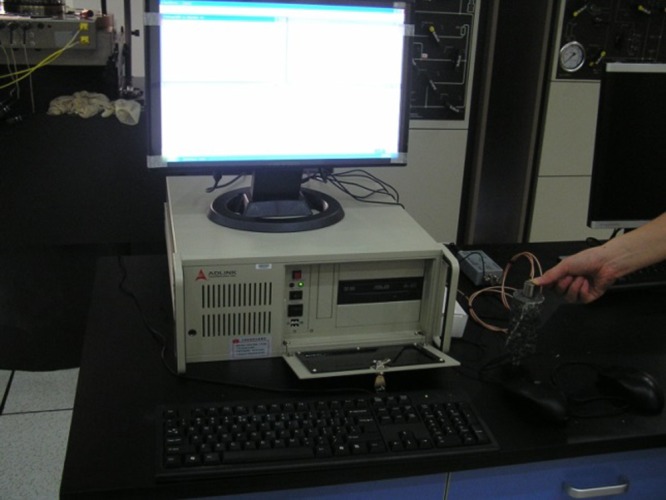
P-wave velocity test of intact rock.

The P-wave velocity of intact rock was measured using the ADLINK acoustic emission test system, which is made in the USA, as shown in figure 5. The formula for determining the P-wave velocity is as follows:

4.3 $$V_{p}=\frac{D}{t_{p}-t_{0}},$$

where *D* is the centre distance between the launching transducer and receiving transducer, *t*_p_ is the propagation time of the P-wave velocity in a rock sample, and *t*_0_ is the zero delay of the instrument system. The mean P-wave velocities of different types of intact rock (C_1d_, C_1b_, C_2w_ and P_1q+m_) were determined to be 5344 m s^−1^, 5130 m s^−1^, 4744 m s^−1^ and 5514 m s^−1^, respectively.

## 5. The analysis of damage index

Determining how to quantify the degree of damage undergone by rock materials that are damaged but not destroyed has been the focus of many studies. As is well known, the strength and P-wave velocity of damaged rock are less than those of intact rock. In this regard, a new method for calculating the damage index *D*_*R*_ was proposed based on the field point load strength test, as expressed in equation (2.4). The damage index *D*_*R*_ can also be expressed in terms of the integrality coefficient *K*_*V*_, as shown in equation (3.11). The integrality coefficient *K*_*V*_ can be defined by the P-wave velocity, as shown in equation (2.3). To verify the accuracy and validity of the new damage index *D*_*R*_, the point load strength and P-wave velocity were measured in the field.

**Figure 6.**
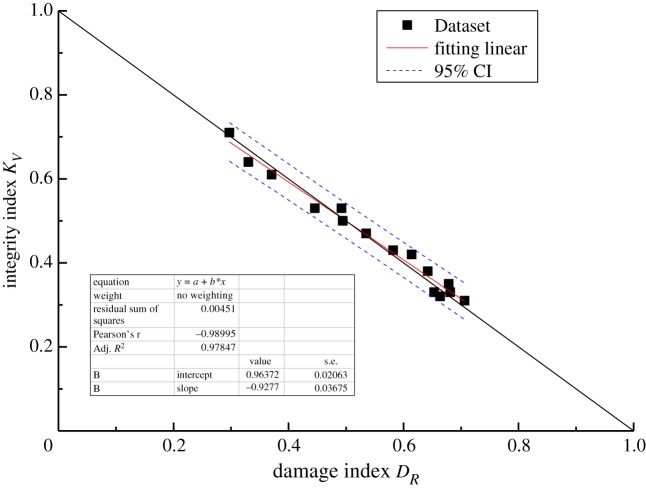
Comparison of the *D*_*R*_ value determined by the new method with the theoretical value for C_1d._

**Figure 7.**
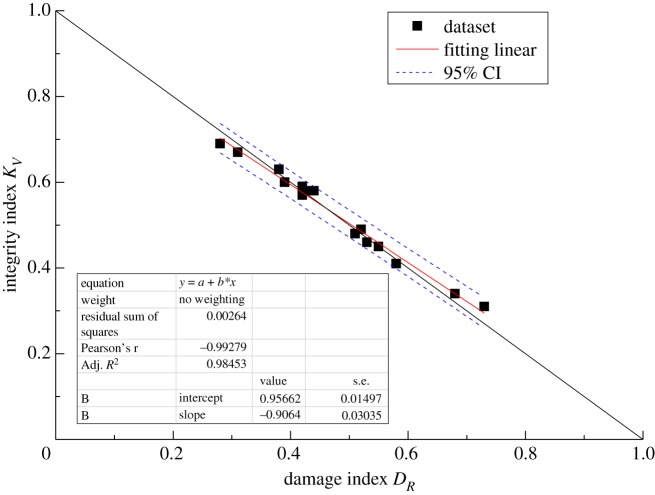
Comparison of the *D*_*R*_ value determined by the new method with the theoretical value for C_1b._

**Figure 8.**
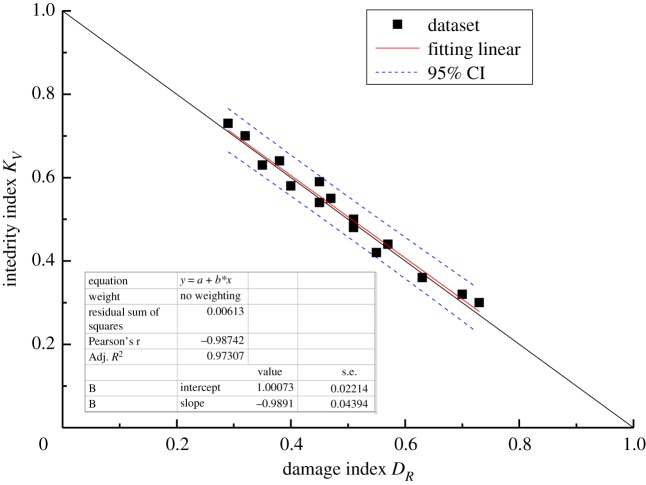
Comparison of the *D*_*R*_ value determined by the new method with the theoretical value for C_2w._

**Figure 9.**
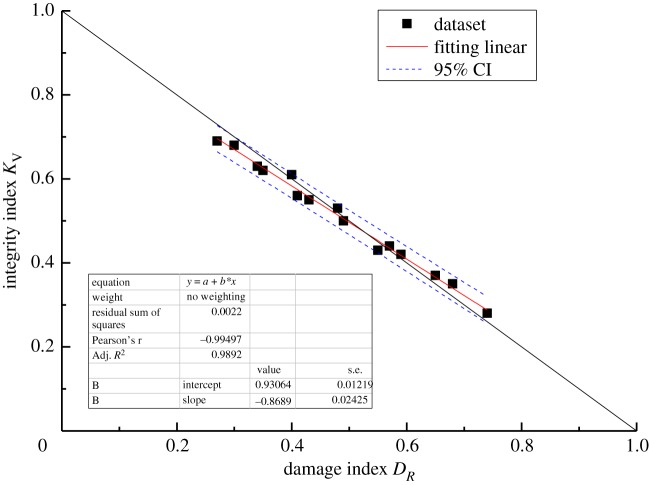
Comparison of the *D*_*R*_ value determined by the new method with the theoretical value for P_1q+m._

The results are listed in table 8. All deviations are less than 10%, except for the one deviation of 11.87%. Moreover, the theoretical correlation between *D*_*R*_ and *K*_*V*_ satisfies the linear relationship established by equation (3.12). Thus, linear regression analysis was used to determine the relationship between *D*_*R*_ and *K*_*V*_, with the confidence limits set to 95%. The purpose of linear regression analysis is to validate the accuracy between a theoretical correlation (in this case, the correlation between *D*_*R*_ and *K*_*V*_) and test results. The linear regression equation takes the form y=a+bx, where *b* is the regression coefficient. The parameter *a* is a constant representing the value of *y* when the independent variable *x* is zero. The cross-correlations of the new damage index *D*_*R*_ and the integrality coefficient *K*_*V*_ for linear regression are shown graphically in figures 6–9. The black solid line is the theoretical line between *D*_*R*_ and *K*_*V*_ according to equation (3.12), the fitting line is the red solid line, and the confidence intervals are indicated by the blue dotted line.

**Table 8.** The calculation results of *D*_*R*_ (AVE---absolute value of relative error).

	C_1d_				C_1b_				C_2w_				P_1q+m_			
rock type	*D*_*R*_	1 − *D*_*R*_	*K*_*V*_	AVE	*D*_*R*_	1 − *D*_*R*_	*K*_*V*_	AVE	*D*_*R*_	1 − *D*_*R*_	*K*_*V*_	AVE	*D*_*R*_	1 − *D*_*R*_	*K*_*V*_	AVE
Test 1	0.37	0.63	0.61	3.14%	0.58	0.42	0.41	2.77%	0.57	0.43	0.44	2.74%	0.34	0.66	0.63	5.27%
Test 2	0.58	0.42	0.43	2.77%	0.28	0.72	0.69	4.90%	0.73	0.27	0.30	8.84%	0.65	0.35	0.37	5.20%
Test 3	0.61	0.39	0.42	8.16%	0.68	0.32	0.34	6.32%	0.35	0.65	0.63	2.44%	0.48	0.52	0.53	2.42%
Test 4	0.65	0.35	0.33	5.07%	0.44	0.56	0.58	4.21%	0.47	0.53	0.55	3.74%	0.35	0.65	0.62	4.27%
Test 5	0.54	0.46	0.49	5.17%	0.55	0.45	0.45	0	0.51	0.49	0.50	1.67%	0.74	0.26	0.28	6.58%
Test 6	0.49	0.51	0.53	4.19%	0.51	0.49	0.48	2.29%	0.29	0.71	0.73	3.38%	0.40	0.60	0.61	1.37%
Test 7	0.68	0.32	0.33	3.40%	0.31	0.69	0.67	3.40%	0.70	0.30	0.32	6.64%	0.30	0.70	0.68	2.45%
Test 8	0.71	0.29	0.31	5.28%	0.39	0.61	0.60	1.44%	0.45	0.55	0.44	2.54%	0.43	0.57	0.55	2.76%
Test 9	0.68	0.32	0.35	8.22%	0.38	0.62	0.63	1.54%	0.40	0.60	0.58	4.15%	0.41	0.59	0.56	4.78%
Test 10	0.30	0.70	0.73	3.71%	0.42	0.58	0.57	2.59%	0.45	0.55	0.59	6.86%	0.59	0.41	0.42	1.23%
Test 11	0.33	0.67	0.64	4.63%	0.43	0.57	0.58	1.22%	0.55	0.45	0.42	6.89%	0.55	0.45	0.43	5.66%
Test 12	0.66	0.34	0.32	5.12%	0.53	0.47	0.46	2.87%	0.63	0.37	0.36	1.98%	0.49	0.51	0.50	1.51%
Test 13	0.49	0.51	0.50	1.09%	0.52	0.48	0.49	2.65%	0.38	0.62	0.64	3.14%	0.57	0.43	0.44	3.28%
Test 14	0.64	0.36	0.38	5.80%	0.42	0.58	0.59	2.20%	0.32	0.68	0.70	2.36%	0.27	0.73	0.69	5.99%
Test 15	0.45	0.55	0.53	4.57%	0.73	0.27	0.31	11.87%	0.51	0.49	0.48	1.92%	0.68	0.32	0.35	7.68%

All of the results demonstrate a reasonable linear relationship with a high coefficient of determination. The correlation coefficients *R*^2^ of rock masses C_1d_, C_1b_, C_2w_ and P_1q+m_ are 0.98, 0.98, 0.97 and 0.99, respectively. Moreover, when the fitting straight line is close and parallel to the theoretical line, the damage index computed using the current method is in good agreement with the damage index computed based on the P-wave velocity. Overall, the results indicate that the current method is effective and can be used to calculate the elastic modulus and unconfined compressive strength of rock masses.

## 6. Discussion

Two failure modes, intact rock failure and splitting along a pre-existing joint, were observed in the field point load strength experiment. The new damage index *D*_*R*_ is defined as a point load strength ratio whose numerator is the strength of splitting along a pre-existing joint and whose denominator is the strength of intact rock failure. The strength ratio is very similar to the damage index of rock masses. Hence, *in situ* tests were designed to verify the newly developed index *D*_*R*_, including an acoustic emission test, a P-wave test and a field point load strength test.

Theoretical derivations indicate that it is feasible to use the point load strength to define the damage index of a rock mass. The theoretical results obtained in this study show that the ratio between the point load strength of splitting along a pre-existing joint and that of intact rock failure is correlated with the integrity coefficient. Typically, the integrity coefficient of a rock mass decreases with increasing joint density, as occurs in the formation of micro-cracks or macroscopic fractures. Hence, the degree of damage sustained by a rock mass must be assessed in the study area. This study considered the point load strength of rock masses undergoing splitting along a pre-existing joint or intact rock failure. However, the degree to which joints developed in the samples and the direction along which they did so could not be accurately measured. We assumed that the splitting of a sample along a pre-existing joint always occurs along the weakest surface. Thus, the newly developed *D*_*R*_ is determined solely by the failure property of field point load strength.

## 7. Conclusion

Based on the statistical results obtained from field point load strength tests, two different failure types were discovered. The specific relationship between the field point load strength ratio *D*_*R*_ and the integrity coefficient *K*_*V*_ of rock masses was determined in this study. To this end, theoretical derivations (§3) and a series of tests (§4) were conducted. Finally, a new method for calculating the damage index in terms of the field point load strength was proposed. Compared with current methods for determining the integrality coefficient, which is the ratio between the P-wave velocity measured in the laboratory and that measured in the field, this new method provides an easier and faster way of estimating the integrality coefficient or the damage index of rock masses. Moreover, the method can be used to estimate the elastic modulus and unconfined compressive strength of rock masses. Therefore, the technique has broad application prospects in underground geotechnical engineering. In conclusion, this new method for calculating the damage index should be further tested and verified by conducting more empirical strength tests, such as those measuring uniaxial compressive strength, uniaxial tensile strength and triaxial compressive strength.

## Supplementary Material

Reviewer comments

## Data accessibility

Our data are deposited at Dryad Digital Repository: http://dx.doi.org/10.5061/dryad.bm76m89 [50].

## References

[1] Deere DU 1967 Technical description of rock cores for engineering purposes. Rock Mech. Rock Eng. 1, 107–116.

[2] Bieniawski ZT 1976 Rock mass classification in rock engineering. In Exploration for rock engineering, Proc. of the Symp., vol. 1 (ed. Bieniawski ZT), pp. 97–106. Rotterdam, The Netherlands: Balkema.

[3] Bieniawski ZT 1989 Engineering rock mass classification: a manual. New York, NY: Wiley.

[4] Barton NR, Lien R, Lunde J 1974 Engineering classification of rock masses for the design of tunnel support. Rock Mech. 6, 189–239. (10.1007/BF01239496)

[5] Barton N 2002 Some new Q-value correlations to assist in site characterization and tunnel design. Int. J. Rock Mech. Min. Sci. 39, 185–216. (10.1016/S1365-1609(02)00011-4)

[6] Hoek E, Kaiser PK, Bawden WF 1995 Support of underground excavations in hard rock. London, UK: Taylor & Francis.

[7] Hoek E, Marinos P, Beniss M 1998 Applicability of the geological strength index (GSI) classification for very weak and sheared rock masses—the case of Athens Schist formation. Bull. Eng. Geol. Environ. 57, 151–160. (10.1007/s100640050031)

[8] Aydan O, Dalgic S 1998 Prediction of deformation behavior of 3-lanes Bolu tunnels through squeezing rocks of North Anatolian fault zone (NAFZ). In Proc. of Regional Symp. on Sedimentary Rock Engineering, Taipei, Taiwan, 20–22 November, pp. 228–233.

[9] Yudhbir WL, Prinzl F 1983 An empirical failure criterion for rock masses. In Proc. of 5th International Society for Rock Mechanics Congress, Melbourne, Australia, 11–15 January, vol. 1, pp. B1–B8.

[10] Laubscher DH 1984 Design aspects and effectiveness of support system in different mining conditions. Trans. Inst. Min. Met. 93, A70–A81.

[11] Singh B, Goel RK, Mehrotra VK, Garg SK, Allu MR 1998 Effect of intermediate principal stress on strength of anisotropic rock mass. Tunnel. Underground Space Technol. 13, 71–79. (10.1016/S0886-7798(98)00023-6)

[12] Ramamurthy T, Rao GV, Rao KS 1985 A strength criterion for rocks. In Proc. of Indian Geotechnical Conf., Roorkee, India, 16–18 December, vol. 1, pp. 59–64.

[13] Ramamurthy T 1996 Stability of rock mass—eighth Indian Geotechnical Society Annual Lecture. Indian Geotech. J. 16, 1–73.

[14] Kalamaras GS, Bieniawski ZT 1993 A rock mass strength concept for coal seams. In Proc. of 12th Conf. on Ground Control in Mining, Morgantown, WV, 3–5 August, pp. 274–283.

[15] Sheorey PR 1997 Empirical rock failure criteria. Rotterdam, The Netherlands: Balkema.

[16] Hoek E, Carranza-Torres C, Corkum B 2002 Hoek–Brown failure criterion—2002 edition. In Proc. of 5th North American Rock Mechanical Symp. and 17th Tunneling Association of Canada Conf.: NARMS-TAC 2002. Mining Innovation and Technology, Toronto, Canada, vol. 1, pp. 267–273.

[17] American Association of State Highway and Transportation Officials (AASHTO). 1996 Standard specifications for highway bridges, 16th edn Washington, DC: AASHTO.

[18] Lian YZ 2010 Estimating the strength of jointed rock masses. Rock Mech. Rock Eng. 43, 391–402. (10.1007/s00603-009-0065-x)

[19] Kulhawy FH, Goodman RE 1987 Foundations in rock. In Ground engineer's reference book (ed. Bell FG), chap. 15. London, UK: Butterworths.

[20] Singh B, Goel RK 1999 Rock mass classifications—a practical approach in civil engineering. Amsterdam, The Netherlands: Elsevier Ltd.

[21] Hoek E, Brown ET 1997 Practical estimates of rock mass strength. Int. J. Rock Mech. Min. Sci. 34, 1165–1186. (10.1016/S1365-1609(97)80069-X)

[22] Gokceoglu C, Sonmez H, Kayabasi A 2003 Predicting the deformation moduli of rock masses. Int. J. Rock Mech. Min. Sci. 40, 701–710. (10.1016/S1365-1609(03)00062-5)

[23] Hoek E, Diederichs MS 2006 Empirical estimation of rock mass modulus. Int. J. Rock Mech. Min. Sci. 43, 203–215. (10.1016/j.ijrmms.2005.06.005)

[24] Coon RF, Merritt AH 1970 Predicting in situ modulus of deformation using rock quality indexes. In Determination of the in situ modulus of deformation of rock, pp. 154–173. ASTM International.

[25] Serafim JL, Pereira JP 1983 Considerations on the geomechanical classification of Bieniawski. In Proc. of Int. Symp. on Engineering Geology and Underground Construction, Lisbon, Portugal, vol. 1, pp. 3–44. Rotterdam, The Netherlands: A.A. Balkema.

[26] Zhang L, Einstein HH 2004 Using RQD to estimate the deformation modulus of rock masses. Int. J. Rock Mech. Min. Sci. 41, 337–341. (10.1016/S1365-1609(03)00100-X)

[27] Bieniawski ZT 1978 Determining rock mass deformability: experience from case histories. Int. J. Rock Mech. Min. Sci. Geomech. Abstr. 15, 237–247. (10.1016/0148-9062(78)90956-7)

[28] Nicholson GA, Bieniawski ZT 1990 A nonlinear deformation modulus based on rock mass classification. Int. J. Min. Geol. Eng. 8, 181–202. (10.1007/BF01554041)

[29] Mitri HS, Edrissi R, Henning J 1994 Finite element modeling of cablebolted stopes in hard rock ground mines. The SME Annual Meeting, Albuquerque, NM, 14–17 February, pp. 94–116.

[30] Ramamurthy T 1993 Strength and modulus response of anisotropic rocks. In Compressive rock engineering: principles, practice and projects, *vol. 1* (ed. Hudson JA), pp. 313–329. Oxford, UK: Pergamon Press.

[31] Singh M, Rao KS 2005 Empirical methods to estimate the strength of jointed rock masses. Eng. Geol. 77, 127–137. (10.1016/j.enggeo.2004.09.001)

[32] Broch E, Franklin JA 1972 The point-load strength test. Int. J. Rock Mech. Min. Sci. Geomech. Abstr. 9, 669–676. (10.1016/0148-9062(72)90030-7)

[33] Bieniawski ZT 1975 The point-load test in geotechnical practice. Eng. Geol. 9, 1–11. (10.1016/0013-7952(75)90024-1)

[34] Tsiambaos G, Sabatakakis N 2004 Considerations on strength of intact sedimentary rocks. Eng. Geol. 72, 261–273. (10.1016/j.enggeo.2003.10.001)

[35] Kahraman S, Gunaydin O, Fener M 2005 The effect of porosity on the relation between uniaxial compressive strength and point load index. Int. J. Rock Mech. Min. Sci. 42, 584–589. (10.1016/j.ijrmms.2005.02.004)

[36] Fener M, Kahraman S, Bilgil A, Gunaydin O 2005 A comparative evaluation of indirect methods to estimate the compressive strength of rocks. Rock Mech. Rock Eng. 38, 329–343. (10.1007/s00603-005-0061-8)

[37] Diyuan L, Louis N, Yuen W 2013 Point load test on meta-sedimentary rocks and correlation to UCS and BTS. Rock Mech. Rock Eng. 46, 889–896. (10.1007/s00603-012-0299-x)

[38] ASTM (American Society for Testing and Materials). 2001 *Standard method for determination of the point load strength index of rock. Designation D*. Vol. 04.08, pp. 5731–5795.

[39] Panek LA, Fannon TA 1992 Size and shape effects in point load tests of irregular rock fragments. Rock Mech. Rock Eng. 25, 109–140. (10.1007/BF01040515)

[40] Kahraman S, Gunaydin O 2009 The effect of rock classes on the relation between uniaxial compressive strength and point load index. Bull. Eng. Geol. Environ. 68, 345–353. (10.1007/s10064-009-0195-0)

[41] The National Standards Compilation Group of People's Republic of China. 1994 GB50218-94 standard for classification of engineering rock masses standard for classification of engineering rock masses. Beijing, People's Republic of China: Planning Press.

[42] Lemaitre J 1984 How to use damage mechanics. Nucl. Eng. Des. 80, 233–245. (10.1016/0029-5493(84)90169-9)

[43] Palmstrom A, Singh R 2001 The deformation modulus of rock masses comparisons between in-situ tests and indirect estimates. Tunnel. Underground Space Technol. 16, 115–131. (10.1016/S0886-7798(01)00038-4)

[44] Khandelwal M, Ranjith PG 2010 Correlating index properties of rocks with P-wave measurements. J. Appl. Geophys. 71, 1–5. (10.1016/j.jappgeo.2010.01.007)

[45] Diamantis K et al. 2011 Correlating wave velocities with physical, mechanical properties and petrographic characteristics of peridotites from the central Greece. Geotech. Geol. Eng. 29, 1049–1062. (10.1007/s10706-011-9436-7)

[46] Yagiz S 2011 Correlation between slake durability and rock properties for some carbonate rocks. Bull. Eng. Geol. Environ. 70, 377–383. (10.1007/s10064-010-0317-8)

[47] Yagiz S 2011 P-wave velocity test for assessment of geotechnical properties of some rock materials. Bull. Mater. Sci. 34, 947–953. (10.1007/s12034-011-0220-3)

[48] Sharma PK, Khandelwal M., Singh TN 2011 A correlation between Schmidt hammer rebound numbers with impact strength index, slake durability index and P-wave velocity. Int. J. Earth Sci. (Geol. Rundsch). 100, 189–195. (10.1007/s00531-009-0506-5)

[49] Martínez-Martínez J, Benavente D, García-del-Cura MA 2012 Comparison of the static and dynamic elastic modulus in carbonate rocks. Bull. Eng. Geol. Environ. 71, 263–268. (10.1007/s10064-011-0399-y)

[50] Wen L, Luo Zq, Yang Sj, Qin Yg, Ma Sw, Jiang H 2019 Data from: A new method for evaluating the rock mass damage index based on the field point load strength *Dryad Digital Repository*. (10.5061/dryad.bm76m89) PMC6458395 31032017

